# Prolyl Oligopeptidase From *Leishmania infantum*: Biochemical Characterization and Involvement in Macrophage Infection

**DOI:** 10.3389/fmicb.2020.01060

**Published:** 2020-05-28

**Authors:** Camila Lasse, Clênia S. Azevedo, Carla N. de Araújo, Flávia N. Motta, Milene A. Andrade, Amanda Pereira Rocha, Iracyara Sampaio, Sébastien Charneau, Marc Gèze, Philippe Grellier, Jaime M. Santana, Izabela M. D. Bastos

**Affiliations:** ^1^Pathogen-Host Interface Laboratory, Department of Cell Biology, University of Brasília, Brasília, Brazil; ^2^UMR 7245 MCAM, Musèum National d’Histoire Naturelle, Centre National de la Recherche Scientifique, Paris, France; ^3^Faculty of Ceilandia, University of Brasília, Brasília, Brazil; ^4^Laboratory of Protein Chemistry and Biochemistry, Department of Cell Biology, University of Brasília, Brasília, Brazil; ^5^CeMIM, Musèum National d’Histoire Naturelle, Paris, France

**Keywords:** leishmaniasis, protease, POPLi, virulence factor, drug target

## Abstract

*Leishmania infantum* is a flagellated protozoan and one of the main causative agents of visceral leishmaniasis. This disease usually affects the human reticuloendothelial system, can cause death and available therapies may lead to serious side effects. Since it is a neglected tropical disease, the incentives for the development of new drugs are insufficient. It is important to know *Leishmania* virulence factors that contribute most to the disease in order to develop drugs. In the present work, we have produced *L. infantum* prolyl oligopeptidase (rPOPLi) in *Escherichia coli*, and investigated its biochemical properties as well as the effect of POP inhibitors on its enzymatic activity and on the inhibition of the macrophage infection by *L. infantum*. The optimal activity occurred at pH 7.5 and 37°C in the presence of DTT, the latter increased rPOPLi catalytic efficiency 5-fold on the substrate *N-*Suc-Gly-Pro-Leu-Gly-Pro-AMC. The enzyme was inhibited by TPCK, TLCK and by two POP specific inhibitors, Z-Pro-prolinal (ZPP, IC_50_ 4.2 nM) and S17092 (IC_50_ 3.5 nM). Besides being a cytoplasmic enzyme, POPLi is also found in punctuate structures within the parasite cytoplasm or associated with the parasite plasma membrane in amastigotes and promastigotes, respectively. Interestingly, S17092 and ZPP prevented parasite invasion in murine macrophages, supporting the involvement of POPLi in the invasive process of *L. infantum.* These data suggest POPLi as a virulence factor that offers potential as a target for designing new antileishmanial drugs.

## Introduction

Leishmaniasis is a group of diseases caused by the *Leishmania* genus, protozoan parasites that have more than 20 infective species to mammals, which are transmitted by the bite of female phlebotomine during blood meal (Nassif et al., 2016). According to the World Health Organization (WHO), leishmaniasis are neglected tropical diseases endemic in 102 countries of American, European, Asian, and African continents, responsible for 1.3 million new cases and approximately 30,000 deaths annually (Organización Panamericana de la Salud [OPS], 2019).

The biological diversity of *Leishmania* can induce different clinical manifestations ranging from skin and mucosal lesions to a more severe systemic form affecting vital organs (World Health Organization [WHO], 2019). Visceral leishmaniasis, also known as kala-azar, caused by *L. donovani* and *L. infantum*, is characterized by hepatosplenomegaly and lymphadenopathy associated to cellular immunity depression and can be fatal if not treated (Choi and Lerner, 2001). The available treatments for leishmaniasis are toxic, long-lasting, expensive and are related to emerging drug resistance (reviewed by Hendrickx et al., 2019). Thus, the identification and characterization of pathogen virulence factors as potential targets for drug and vaccine development is a rational strategy in prospection of accessible and efficient therapies for these diseases.

Proteases play crucial roles in both physiology and infectivity of pathogens and arise as potential drug targets (Rao et al., 1998). Among them, prolyl oligopeptidase (POP, EC 3.4.21.26) is an ubiquitous serine-protease (Kuwabara, 1992; Kocadag Kocazorbaz and Zihnioglu, 2017) belonging to the S9 family (Rawlings et al., 2018) that cleaves mainly peptides up to 30 amino acids in the carboxyl side of proline and, less efficiently, alanine residue. In humans, POPs are involved in inflammatory responses (Kumar et al., 2016), angiogenesis (Myöhänen et al., 2011), and regulation/maturation of peptide hormones and neuropeptides. Abnormal POP activity levels were reported in neural tissue or in plasma of individuals suffering of amnesia, depression, Alzheimer’s disease, and bipolar disorder (Laitinen et al., 2001; Männisto et al., 2007; Svarcbahs et al., 2019).

In mammal pathogens, POPs have also been identified as potential virulence factors. *Mycobacterium tuberculosis* POP induces the secretion of pro-inflammatory Th1 cytokines as TNF, IL-12p70, IL-6, IL-23, and IL-1b, modulating murine macrophages and suggesting its involvement in *M. tuberculosis* infectivity (Portugal et al., 2017). Amongst Trypanosomatidae, *Trypanosoma cruzi* POP (POPTc80) is mainly secreted by infective trypomastigote forms capable to hydrolyse extracellular matrix proteins type I and IV collagens and fibronectin *in vitro* and to degrade *in situ* rat mesentery collagen fibers (Santana et al., 1997; Grellier et al., 2001). It was suggested that POPTc80 could facilitate the host cell infection through collagen fiber degradation in extracellular matrix and basement lamina enabling parasite access to host cell surface. Its potential role in infection was assessed by several specific inhibitors, developed by combinatory chemistry (Vendeville et al., 1999a, b, 2002; Joyeau et al., 2000; Grellier et al., 2001; Bal et al., 2003), capable to block non-phagocytic host cell invasion by trypomastigotes in a dose-dependent manner with a significant selectivity *versus* human POP (Ki values 60–fold lower). Reproducible results have been achieved with Tulahuen, Y and Berenice *T. cruzi* strains (Grellier et al., 2001). New POPTc80 inhibitors were obtained by virtual-screening showing an innovative way for drug development against Chagas disease (de Almeida et al., 2016). Recently, POPTc80 was proposed as an antigen for vaccine development against *T. cruzi* infection (Bivona et al., 2018). Immunized mice with recombinant POPTc80 elicited strong cell-mediated immunity, showed a decreased parasitemia and a higher survival rate compared with non-immunized mice after trypomastigote challenge. During the chronic phase of the infection, they presented lower levels of myopathy-linked enzymes, parasite burden, electrocardiographic disorders and inflammatory cells. Moreover, immunoprotection was extended to *T. cruzi* strains from different discrete typing units (DTUs) (Bivona et al., 2018).

Likewise, *Trypanosoma brucei* POP (POPTb) also hydrolyses purified type I human collagen and mesenteric stretched collagen fibers, what might facilitate parasite penetration through blood and lymphatic vessel endothelium as well as blood-brain barrier (Bastos et al., 2010). POPTb is detected in the plasma of *T. brucei*-infected mice and remains active proportionally to parasitemia peaks. In addition, POPTb degrades host hormones and neurotransmitter peptides (GnRH, TRH, neurotensin, endorphin, bradykinin), which could play a role in neuroendocrine disturbances (Ndung’u et al., 1992; Tetaert et al., 1993; Bastos et al., 2010), since abnormal levels of GnRH and TRH seem to correlate with hypogonadism and hypothyroidism, symptoms associated to sleeping sickness (Hublart et al., 1988). It has been showed that POPTb (Bastos et al., 2010) and pyroglutamyl peptidase (TbPGP) (Morty et al., 2006) generate oligopeptide signals, which once received via *Tb*GPR89 transporter regulate stumpy formation. This signalization was proposed as a trypanosome quorum sensing mechanism to control virulence and infection spread (Rojas et al., 2019). In *Trypanosoma evansi* infections, mice inoculated with a null mutant clone for a POP-like gene were able to survive longer than those inoculated with wild-type parasites (Kangethe et al., 2017). As well, *Schistosoma mansoni* POP may contribute to parasite survival by cleaving host bioactive peptides (Fajtová et al., 2015). More recently, the inhibition of *Setaria cervi* (a bovine filarial worm) POP by *Z*-Pro-prolinal induced Ca^2+^ signaling, via phospholipase C stimulation, causing mitochondrial mediated apoptosis in the parasite (Wadhawan et al., 2018).

The overall picture that emerges from these studies suggests POPs are potential therapeutic targets against parasites (Bastos et al., 2013). In this work, we investigated *L. infantum* POP (POPLi) biochemical properties and its importance to macrophage infection by *L. infantum*. We found that treatment with specific POP inhibitors, *Z*-Pro-Prolinal or S17092, results in diminished macrophage infection, suggesting that POPLi may be a key player in host cell infection.

## Materials and Methods

### Parasites

*Leishmania (L.) infantum* (MHOM/BR/2002/LPC-RPV) promastigotes were maintained in Schneider’s medium supplemented with 10% (v/v) fetal bovine serum (FBS, Gibco) and 100 μg/mL gentamicin at 28°C. Axenic amastigotes were obtained by promastigotes incubation in M199 medium at pH 5.4 supplemented with 10% FBS and 100 μg/mL gentamicin at 37°C with 5% CO_2_ for 3 days (Moreno et al., 2011).

### POPLi Heterologous Expression

The full-length *popli* gene from *L. infantum* (LinJ.36.7060) was synthesized after codon optimization and cloned into the pET-15b plasmid by GenScript (New Jersey, United States). The construction was used to transform *Escherichia coli* BL21(DE3)-AI and recombinant POPLi expression was induced with 0.5 mM isopropylthio-β-D-galactoside (IPTG) and 0.2% L-arabinose at 20°C for 4 h. Subsequently, cells were harvested, lysed with BugBuster^TM^ (Novagen) and soluble extract was cleared by centrifugation at 2,000 ×*g* at 4°C for 10 min. Then, the extract was submitted to a nickel-agarose affinity chromatography, washed with 30 column volumes of 50 mM Tris-base pH 8.0, 500 mM NaCl and 15 mM imidazole, and eluted with 50 mM Tris pH 8.0, 500 mM NaCl and 80 mM imidazole. Purified recombinant protein was analyzed by 10% SDS-PAGE stained with Coomassie Blue and stored in 50% glycerol at −20°C.

### Enzymatic Assays

#### Substrate Specificity

rPOPLi (40 ng/μL) substrate specificity was explored using the following fluorogenic 7-amino-4-methyl coumarin (AMC) substrates Pro-AMC, *N*-Ile-Ala-AMC, Suc-Gly-Pro-AMC, Ala-Ala-Phe-AMC, Arg-Arg-AMC, Gly-Pro-AMC and *N*-Suc-Gly-Pro-Leu-Gly-Pro-AMC (20 μM final concentration). Tests were performed with 25 mM HEPES, 150 mM NaCl and 5 mM DTT at pH 7.5 in a final volume of 100 μL in 96-well plate. The AMC fluorescence releasing was recorded by its emission at 460 nm upon excitation at 380 nm using a microplate fluorescence reader (SpectraMax^®^ M5 – Molecular Devices). All enzymatic assays in this work were performed in triplicate.

#### pH Dependence

To determine the effect of pH on rPOPLi (40 ng/μL) activity, the enzyme activity was assayed over a pH range (5.0, 6.0, 7.0, 7.5, 8.0, 8.5, 9.0, and 10.0) using 20 μM *N*-Suc-Gly-Pro-Leu-Gly-Pro-AMC in AGMT buffer (25 mM acetic acid, 25 mM glycine, 50 mM MES, 75 mM Tris, 1 mM EDTA, and 1 mM DTT) adjusted to different pH values (Fülöp et al., 1998).

#### Optimal Buffer

rPOPLi (40 ng/μL) was tested in two different buffers, 50 mM Tris or 25 mM HEPES, both at pH 7.5, using 20 μM *N*-Suc-Gly-Pro-Leu-Gly-Pro-AMC, in the presence or the absence of additives (NaCl and DTT). The AMC fluorescence releasing was recorded as explained above.

#### Temperature

To determine the effect of temperature on rPOPLi (40 ng/μL) activity, assays were performed at 28, 37, 40, and 45°C. Recombinant POPLi was pre-incubated for 5 min at each temperature before addition of *N*-Suc-Gly-Pro-Leu-Gly-Pro-AMC (20 μM final concentration) followed by incubation for 20 min. The reaction was stopped with 100 μL of absolute ethanol and transferred to a 96-well plate. The AMC fluorescence releasing was recorded in endpoint mode.

#### Enzymatic Parameters

Different concentrations (6.25 – 200 μM) of *N*-Suc-Gly-Pro-Leu-Gly-Pro-AMC were tested in 25 mM HEPES, 150 mM NaCl, pH 7.5 with 40 ng/μL of rPOPLi with or without 5 mM DTT in order to determine *K*_*m*_ and *K*cat. The values were determined using non-linear regression with GraphPad Prism.

#### Inhibition Tests

The following protease inhibitors 4-(2-Aminoethyl) benzenesulfonyl fluoride hydrochloride (AEBSF, 100 μM), ethylenediamine tetraacetic acid (EDTA, 1 mM), *N-p*-Tosyl-L-phenylalanine chloromethyl ketone (TPCK, 100 μM), *trans*-epoxysuccinyl-L-leucylamido (4-guanidino) butane (E-64, 1 mM), *N-*α-Tosyl-L-lysine chloromethyl ketone hydrochloride (TLCK, 100 μM), Leupeptin (100 μM), Pesptatin (100 μM); or POP specific inhibitors *Z*-Pro-Prolinal (ZPP, 0.3–10 nM) and (2S,3aS,7aS)-1-(((R,R)-2-Phenylcyclopropyl)carbonyl)-2- ((thiazolidin-3-yl)carbonyl)octahydro-1H-indole (S17092, 0.3– 10 nM); or the antiserum directed against rPOPLi (1:50, 1:100, 1:200, 1:400, 1:800, and 1:1600, raised as described below) were pre-incubated for 30 min with 40 ng/μL rPOPLi diluted in 25 mM HEPES at pH 7.5. The reactions were performed after addition of the substrate *N*-Suc-Gly-Pro-Leu-Gly-Pro-AMC (20 μM final concentration). To demonstrate that ZPP and S17092 can penetrate parasite membrane, *L. infantum* promastigotes were incubated in the presence of 1, 10, or 50 μM of either ZPP or S17092 in RPMI medium for 1 h. After that, parasites were washed three times with PBS to remove inhibitors and lysed by three cycles of freezing/thawing. Protein total extract was tested against 20 μM *N*-Suc-Gly-Pro-Leu-Gly-Pro-AMC, Gly-Pro-AMC, Suc-Leu-Leu-Val-Tyr-AMC, and Z-Gly-Gly-Arg-AMC as described above.

### Evaluation of POPLi Activity in Parasite Extracts

*Leishmania infantum* promastigotes and amastigotes were washed three times with PBS (1,000 × *g*, 10 min, 4°C) and resuspended in 500 μL of 25 mM HEPES pH 7.5. Lysis was performed by using BugBuster reagent (Novagen) (total parasite extract – Total), or by three cycles of freezing/thawing. For the latter, soluble (cytoplasmic – CTPL) and insoluble fractions were obtained by centrifugation (16,000 × *g*, 10 min, 4°C). The insoluble fraction was reconstituted in 25 mM HEPES pH 7.5 and 0.1% Triton X-100, centrifuged again to enrich the membrane protein fraction (supernatant, membrane – MEMB) and the cytoskeleton fraction (pellet, cytoskeleton – CSKL) (Kuroda and Porter, 1987), which was reconstituted in 25 mM HEPES pH 7.5. Activity was measured as described above.

### Anti-rPOPLi Serum Production and Western Blotting

Three male BALB/c mice were immunized with four biweekly boosts of 10 μg purified rPOPLi, in which the first boost was emulsified in complete Freund’s adjuvant, followed by two in incomplete Freund’s adjuvant and the last without adjuvant. Sera were collected after the last immunization, diluted 1:1 (v/v) in glycerol, aliquoted and stored at −20°C. All animal experiments were conducted in accordance with Animal Ethics Committee from the University of Brasília guidelines (27764/2016).

*Leishmania infantum* promastigotes (1 × 10^7^ cells) were used to analyze the protein level of POPLi in total parasite extract, cytoplasmic, membrane or cytoskeleton fractions obtained as described above. Purified rPOPLi and parasite fractions were applied on 10% SDS-PAGE and transferred onto nitrocellulose membranes (Amersham Biosciences). After blocking non-specific sites with 5% skimmed milk in Tris-Buffered-Saline containing 0.1% Tween 20 (TBS-T) for 1 h at room temperature, membranes were incubated 1 h with anti-rPOPLi serum (1:200) or anti-His-tag (1:1,000), followed by horseradish peroxidase-conjugated secondary antibody (1:30,000) for 1 h. Immunoreactive bands were detected using Amersham^TM^ ECL^TM^ Prime Western Blotting Detection Reagent (GE Healthcare). As sample loading control, the membrane was probed with the anti-alpha tubulin TAT1 monoclonal antibody IgG2b (1:2,000), kindly provided by Keith Gull (Woods et al., 1989).

### Immunocytolocalization of POPLi

*Leishmania infantum* promastigotes and amastigotes were washed in Phosphate-Buffered Saline (PBS), fixed in 1.0% formaldehyde for 30 min, permeabilized with 0.1% Triton X-100 and after 10 min, glycine was added to 0.1 M and incubated for 10 min. After centrifugation, parasites were resuspended in PBS and spread on glass slides coated with poly-L-lysine (Sigma). Cells were blocked in PBS with 0.1% Triton X-100 and 0.1% BSA for 15 min. Cells were then treated with pre-immune serum (1:100) or anti-POPLi serum (1:100) for 1 h at room temperature, washed three times with PBS for 10 min and stained with Alexa Fluor 488-labeled secondary antibodies (1:200) for 1 h. Five μg/mL DAPI (Sigma) were added for 10 min. Slides were washed with PBS and mounted with Fluorescent Mounting Medium (DAKO). The analysis was carried out using airyscan mode in the confocal fluorescence microscope ZEISS LSM 800. Images were processed using the Fiji software (Schindelin et al., 2012).

### Effect of POP Inhibitors on *Leishmania infantum* Viability

To evaluate the effect of POP inhibitors on parasite viability, 4 × 10^6^ promastigotes/mL were incubated in the presence of different concentrations of S17092 or ZPP (100 μM – 12.5 μM) or 0.1% DMSO (control) for 48 h at 28°C to a final volume of 200 μL in 96-well microplate. Next, 20 μL of resazurin solution (0.39 mM) were added to each well, incubated for 4 h and then fluorescence was recorded (570 nm_*ex*_/595 nm_*em*_) on microplate reader SpectraMax M5 (Molecular Devices, Sunnyvale, CA, United States). The assay was performed in triplicate (Andrade et al., 2016).

### Inhibition of Macrophage Infection by *Leishmania infantum*

Bone marrow-derived macrophages (BMM) were obtained from femur and tibia of BALB/c mice. Briefly, hind legs were gently smashed with RPMI 1640 in a sterile mortar using a pestle. Bone marrow was filtered through a 70 μm cell strainer (Sigma Aldrich) and then centrifuged at 400 × *g* for 10 min at 4°C. Bone marrow was resuspended in complete RPMI 1640 medium (20% FBS and 100 μg/mL gentamycin sulfate) and cells counted in Neubauer chamber by Trypan Blue viability test. Cells were seeded at 2 × 10^5^ cells/mL in 10 mL of complete RPMI 1640 medium with 10 ng/mL murine M-CSF (Peprotech) in Petri dishes at 37°C under 5% CO_2_ for 8 days with re-feeding at day 4 by addition of 10 mL complete RPMI 1640 supplemented with M-CSF. BMM purity was analyzed by phenotypic expression of specific macrophage markers (F4/80 and CD11b, Biolegend) using flow cytometer (BD FACSVerseTM, BD Biosciences) (adapted from Trouplin et al., 2013).

For *in vitro* infection, BMM were harvested and 4 × 10^5^ cells were seeded in 24-well plates, at 37°C, under 5% CO_2_ for 24 h. Infection assays were performed with *L. infantum* at a 10:1 parasite-to-macrophage ratio using infective promastigotes obtained after two successive infections in mice. After parasite addition, plates were quickly centrifuged at 300 × *g* for 20 s and infection followed for 2 h, at 37°C, under 5% CO_2_. Afterward, wells were washed with PBS to eliminate extracellular parasites. Infection rate (% of infected macrophage) was determined by panoptic staining (Instant Prov, Newprov) and counting 100 macrophages under light microscopy (Cunha-Júnior et al., 2017). Different conditions were tested. (1) Infection assay of macrophages by promastigotes preincubated with POP inhibitors: Promastigotes were preincubated 1 h with specific POP inhibitors, ZPP or S17092, at three different concentrations, 1, 10, and 50 μM, or 0.1% DMSO as control, before addition to the BMM monolayers; (2) Infection assay of preincubated macrophages with POP inhibitors: BMM were preincubated 1 h with 50 μM ZPP or S17092, or 0.1% DMSO as control, washed three times with PBS before addition of untreated promastigotes to proceed to the infection assay*;* (3) Infection assay in the presence of rPOPLi (10 μg per well, boiled for 5 min or not boiled); (4) Infection assay of macrophages by promastigotes preincubated with anti-rPOPLi serum: Promastigotes were preincubated 30 min with anti-rPOPLi serum (1:100 and 1:200) before addition to the BMM monolayers; and (5) Infection assay in the presence of POP immune complexes: 10 μg rPOPLi were incubated with anti-rPOPLi serum (1:50, 1:100, and 1:200) for 30 min to form immune complexes. Then, these immune complexes were added along with the parasites to the BMM monolayers. Assays were performed in triplicate and the infection was evaluated by percentage of infected cells compared to the control.

### Statistical Analysis

Graphics were generated using GraphPad Prism 6 software. Results were expressed as mean ± standard error of the mean (SEM) and statistically analyzed with analysis of variance (ANOVA) followed by *t*-Student test. The *p* significance level was broken down in the results.

## Results

### POPLi Enzymatic Properties

**FIGURE 1:**
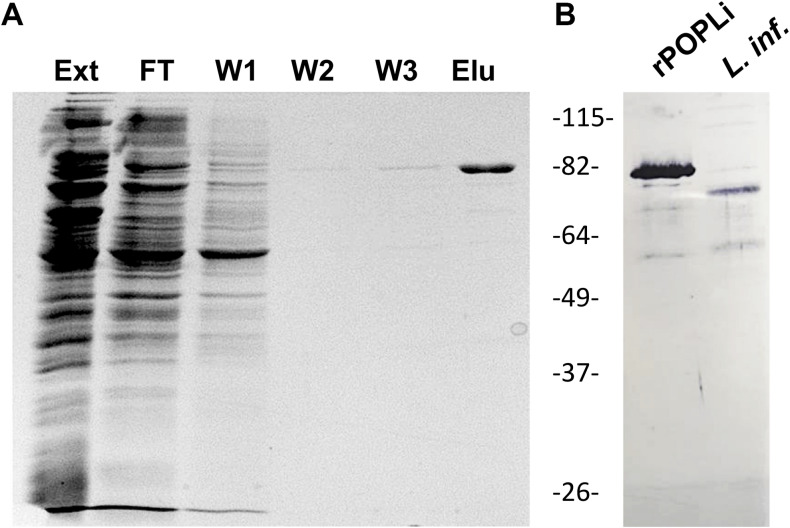
SDS-PAGE and western blot of purified rPOPLi. Analysis of recombinant POPLi expressed in *E. coli.* **(A)** SDS-PAGE of fractions from flow-through, wash and elution steps (12 μL/lane). The gel was subjected to Coomassie Blue staining. Ext, total extract; FT, flow through; W1-W3, washes; Elu, elution fraction. **(B)** Western blot of purified rPOPLi (30 ng) and total protein extracted from 5 × 10^6^ *L. infantum* promastigotes, obtained after freezing and thawing, were performed with the anti-rPOPLi serum (1:200). POPLi predicted molecular weight is ∼78 kDa, and rPOPLi is ∼80 kDa.

After purification by affinity chromatography, the rPOPLi migrated at the expected molecular mass of 80 kDa in SDS-PAGE (Figure 1A). Recombinant POPLi was used to produce antibodies in mice, which cross-reacted with both the recombinant and native protein (78 kDa) from the promastigote extract (Figure 1B).

**FIGURE 2:**
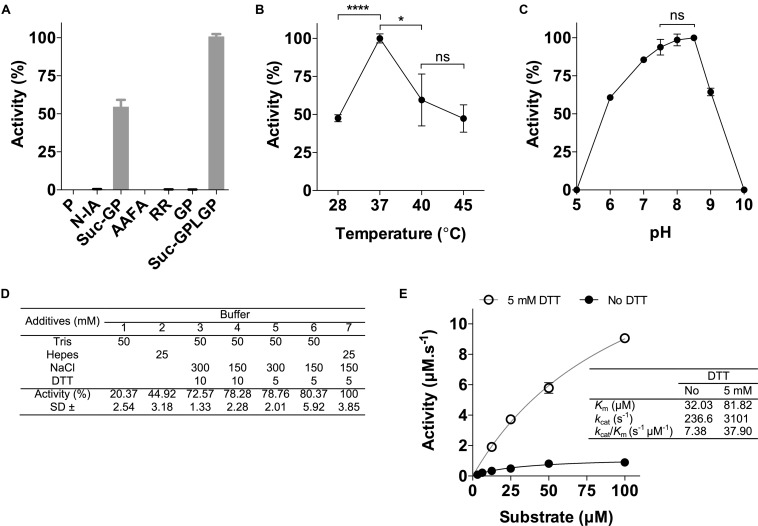
Effects of different substrates, pH values, temperatures, additives and anti-POPLi antibodies on rPOPLi activity. **(A)** Substrates (20 μM final concentration) were tested in 25 mM HEPES pH 7.5 containing 150 mM NaCl and 5 mM DTT. Substrates: P (Pro-AMC), N-IA (*N*-Ile-Ala-AMC), Suc-GP (Suc-Gly-Pro-AMC), AAFA (Ala-Ala-Phe-AMC), RR (Arg-Arg-AMC), GP (Gly-Pro-AMC) and Suc-GPLGP (*N*-Suc-Gly-Pro-Leu-Gly-Pro-AMC). **(B)** Effect of temperature on rPOPLi activity. Activities were determined in pH 7.5 with incubation temperatures ranging from 28 to 45°C. **(C)** Stability of rPOPLi at different pH values. Activities were determined in AGMT buffer adjusted to different pHs. **(D)** Effect buffer (HEPES 25 mM or Tris 50 mM), salt (0, 150, or 300 mM NaCl) and reducing agent DTT (0, 5, or 10 mM) on rPOPLi activity. **(E)** Substrate concentration on rPOPLi activity and determination of enzyme kinetic parameters in the presence or not of DTT. The assays were performed in triplicate. *p < 0.05 and ****p < 0.0001.

The rPOPLi was initially tested in enzymatic assays using the substrate Suc-Gly-Pro-Leu-Gly-Pro-AMC, which is already known to be effectively cleaved by POPTc80 and POPTb (Santana et al., 1997; Bastos et al., 2010). Different fluorogenic substrates were tested to assess the enzyme substrate specificity. rPOPLi showed the highest activity toward Suc-Gly-Pro-Leu-Gly-Pro-AMC and in a less extend, Suc-Gly-Pro-AMC. No hydrolysis was measured with the N-terminal-unblocked substrates Gly-Pro-AMC and Pro-AMC, as well with the other substrates tested (Figure 2A). Suc-Gly-Pro-Leu-Gly-Pro-AMC was therefore used for all the enzymatic assays. In regard to temperature, rPOPLi showed a significantly higher activity at 37°C (Figure 2B) that decreased to 60% at 40°C, and to approximately 45% at 28°C and 45°C. The optimum pH activity is dependent on a slightly alkaline pH, between 7.5 and 8.5 (Figure 2C). It retained approximately 75% activity at pH 7 and approximately 60% at pH 6 and 9. The effects of NaCl and the reducing agent DTT were examined using 50 mM Tris pH 8.5 or 25 mM HEPES pH 7.5. Addition of reducing agent clearly improved the rPOPLi activity. HEPES buffer containing 150 mM NaCl and 5 mM DTT was found optimal for the rPOPLi activity (Figure 2D) and was used to further determine enzymatic parameters. Calculated *K*_*m*_ values were of 32.03 μM in the absence and 81.82 μM in the presence of DTT. Despite *K*_*m*_ augmentation in the presence of DTT, this additive elevated the enzyme catalytic efficiency 5-fold compared to that observed in its absence (Figure 2E).

**FIGURE 3:**
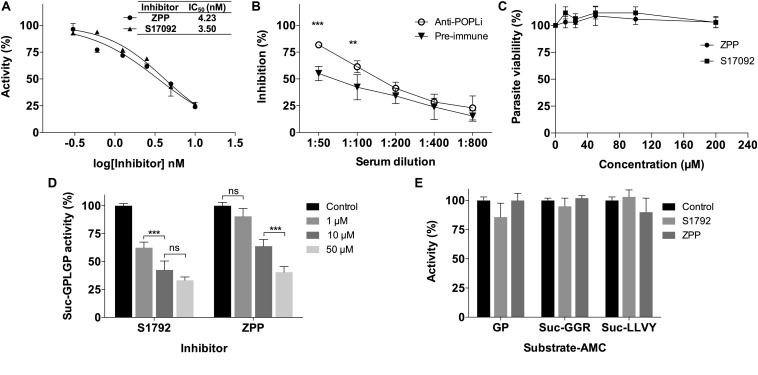
*Leishmania infantum* viability was not affected by POPLi inhibition. **(A)** POPLi inhibition by specific POP inhibitors ZPP and S17092. **(B)** POPLi inhibition by anti-POPLi serum. **(C)** Effect of POP inhibitors on *L. infantum* viability. Promastigotes (4 × 10^6^ cells/mL) were incubated for 48 h in the presence of 12.5 to 200 μM ZPP or S17092, or 0.1% DMSO (control). Viability was determined using Resazurin. Error bars represent standard deviations of triplicate samples. **(D)** ZPP and S17092 can penetrate *L. infantum* cell membrane. Promastigotes were incubated in the presence of either ZPP or S17092 for 1 h. Parasites were washed with PBS and lysed by freezing/thawing. Protein total extract was tested against *N*-Suc-Gly-Pro-Leu-Gly-Pro-AMC. **(E)** Enzymatic activity of other proteases is not affected by ZPP or S17092. Promastigote cell lysate was tested against Gly-Pro-AMC (dipeptidyl peptidases), *Z*-Gly-Gly-Arg-AMC (oligopeptidase B) and Suc-Leu-Leu-Val-Tyr-AMC (cathepsin B). **p < 0.01 and ***p < 0.001.

The activity of rPOPLi was inhibited by TLCK and TPCK (trypsin and chymotrypsin-like protease inhibitors), by 96.8 and 92.3%, respectively at the concentration of 0.1 mM. Pepstatin A (0.1 mM), an aspartic protease specific inhibitor, and AEBSF (10 mM), a serine protease inhibitor, showed weak inhibition activities. The other protease inhibitors had no significant inhibitory activity at the concentrations tested. The POP specific inhibitor, Z-Pro-Prolinal, a modified canonical peptidomimetic compound with high specificity to the active site of POP (Fülöp et al., 1998), inhibited the rPOPLi activity of 81% at 10 nM and resulted in an IC_50_ value of 4.23 nM. For the S17092 POP specific inhibitor, the IC_50_ was 3.50 nM (Figure 3A). The antiserum produced against rPOPLi showed a significant inhibition of the enzyme activity until 1:100 serum dilution (Figure 3B). The observed pre-immune serum inhibition maybe due to immunoglobulins and/or other serum proteins that could weakly interact with POPLi surface, and sterically impair the substrate access to the catalytic site. In addition, neither ZPP nor S17092 affected *L. infantum* promastigote growth in an *in vitro* viability assay after 48h incubation (Figure 3C). Both ZPP and S17092 show effectiveness in penetrating parasite membrane, binding specific to POPLi and inhibiting its enzymatic activity when directly tested in promastigote pre-incubated with the inhibitors before cell lysis (Figure 3D). POP activity decreases in a dose response way with more than 50% of inhibition in the presence of 50 μM ZPP or S17092 (Figure 3D). Lastly, to verify if enzymatic activity of other proteases would be affected by these inhibitors, promastigote cell lysate was tested against Gly-Pro-AMC, Z-Gly-Gly-Arg-AMC and Suc-Leu-Leu-Val-Tyr-AMC, recognized substrates of dipeptidyl peptidases, oligopeptidase B and cathepsin B, respectively (Figure 3E). No significant inhibition was observed even in the highest concentration tested, endorsing the specificity of ZPP and S17092 for POPs.

### POPLi Cytolocalization

**FIGURE 4:**
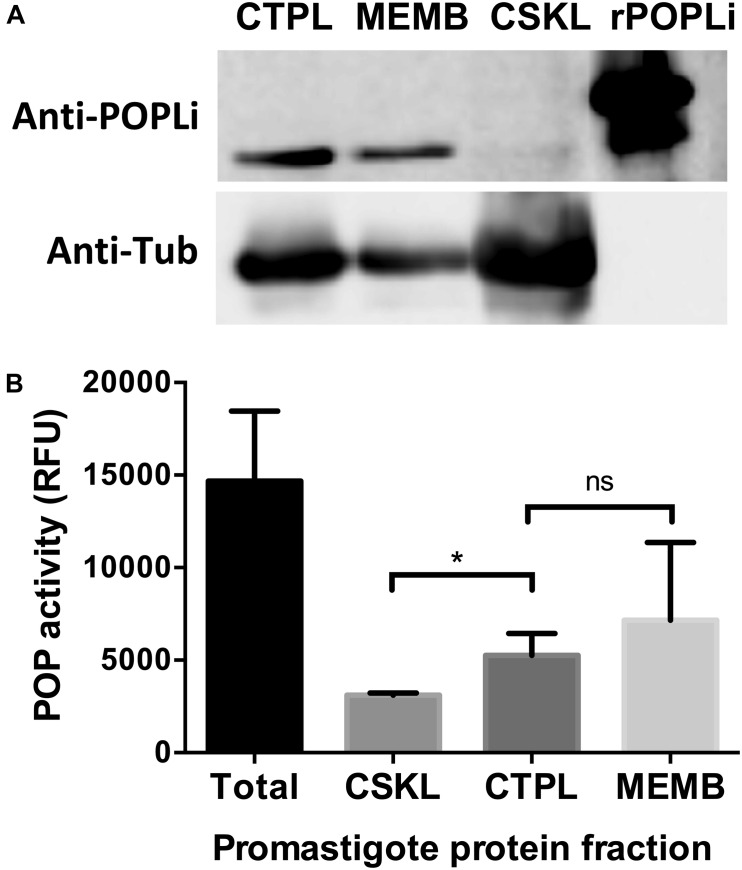
POPLi localization and enzymatic activity in parasite extract fractions. **(A)** 1 × 10^7^ *L. infantum* promastigotes were lysed by freeze and thaw cycles to produce a soluble cytoplasmic fraction (CTPL) and an insoluble fraction, which was solubilized with Triton X-100 to produce an insoluble cytoskeleton fraction (CSKL) and a soluble membrane bound protein fraction (MEMB). Native POPLi expression in all subcellular fractions was analyzed by Western blotting using anti-rPOPLi serum followed by ECL detection. rPOPLi (80 ng) was loaded for reference. As sample loading control, the same membrane was probed with the anti-alpha tubulin TAT1 monoclonal antibody IgG2b (1:2000). **(B)** Enzymatic activity against Suc-Gly-Pro-Leu-Gly-Pro-AMC was measured in total fraction after BugBuster lysis (Total) and in the CTPL, CSKL and MEMB fractions obtained after freezing and thawing lysis. *p < 0.05, Student’s *t*-test.

**FIGURE 5:**
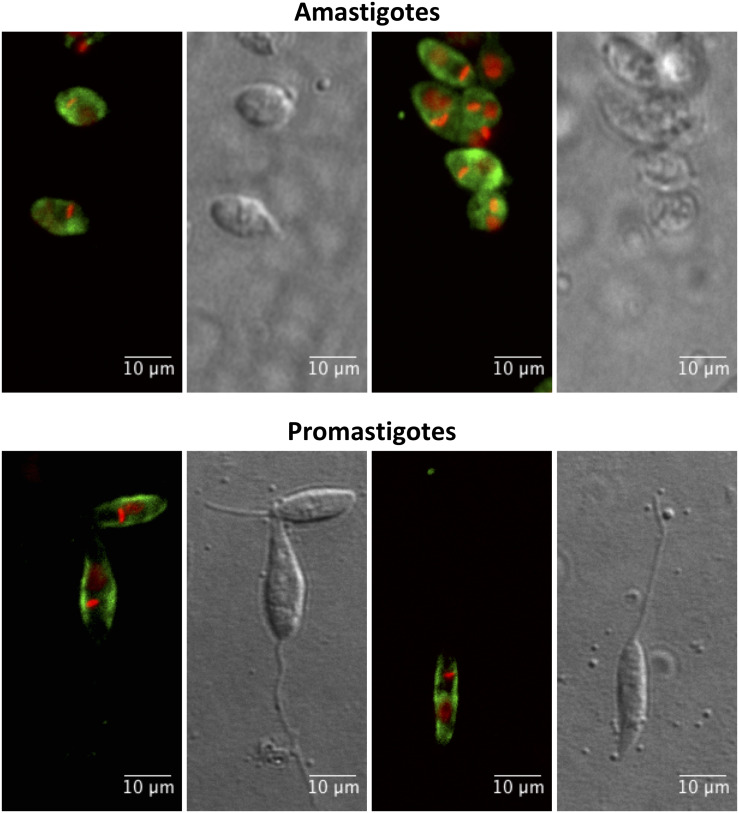
Cellular distribution of POPLi in the developmental forms of *Leishmania infantum.* Axenic amastigotes and promastigotes were fixed in 1% formaldehyde/PBS and stained with POPLi antiserum followed by incubation with Alexa Fluor 488-conjugated anti-mouse IgG (green). Parasite DNA was stained with DAPI (red). Overlay of fluorescence images.

In order to obtain different cellular fractions from the parasite, promastigotes were lysed by freeze and thaw cycles to produce a soluble cytoplasmic fraction (CTPL) and an insoluble fraction, which was solubilized with Triton X-100 to produce an insoluble cytoskeleton fraction (CSKL) and a soluble membrane-bound protein fraction (MEMB). By Western blotting, POPLi was found associated with the cytoplasm and membrane-bound protein fractions (Figure 4A). The highest enzymatic activities against Suc-Gly-Pro-Leu-Gly-Pro-AMC were also measured in these fractions (Figure 4B). Immunofluorescence assays performed on *L. infantum* promastigotes and axenic amastigotes using anti-rPOPLi serum confirmed the enzyme expression in both forms. POPLi was observed in punctuate structures within the cytoplasm of both forms and also concentrated near the cellular membrane from promastigotes, suggesting that POPLi may be also associated with membrane structures or plasma membrane (Figure 5).

### POPLi Inhibition Decreases Macrophage Infection by *Leishmania infantum*

**FIGURE 6:**
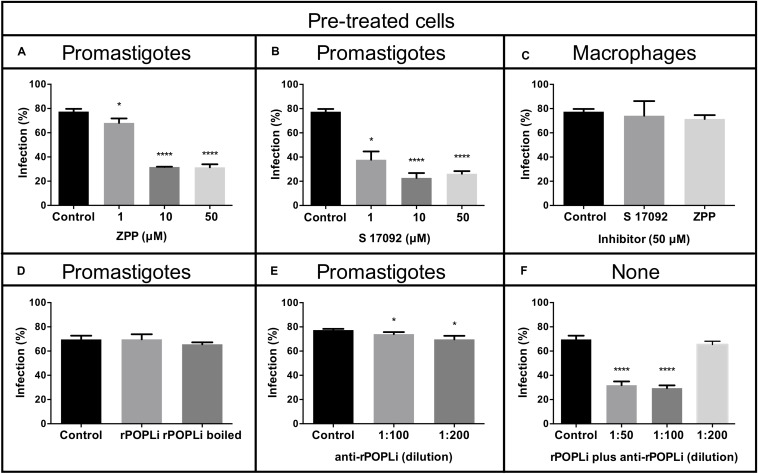
POP specific inhibitors reduce macrophage infection by *Leishmania infantum*. Murine macrophages were infected with *L. infantum* promastigotes (MOI = 10:1) after parasite incubation with POP inhibitors **(A)** Z-Pro-Prolinal (ZPP) and **(B)** S17092. **(C)** Murine macrophages were pre-incubated 1 h with POP inhibitors before infection. **(D)** Infection assay in the presence of rPOPLi (boiled or not boiled). **(E)** Infection assay after 30 min treatment of promastigotes with anti-rPOPLi serum. **(F)** Infection assay in the presence of POP immune complexes: rPOPLi (10 μg) and anti-POPLi antiserum pre-incubation for 30 min to form immune complexes that were added along with the parasites in the infection assay. None: neither promastigotes nor macrophages were submitted to any pre-treatment. Assays were performed in triplicate. Infection was evaluated by percentage of infected cells compared to the control. *p < 0.05 and ****p < 0.0001.

To examine POPLi involvement in murine macrophage infection by *L. infantum*, promastigotes were preincubated 1 h with POP specific inhibitors, ZPP and S17092 before the invasion assay in the presence of inhibitor. A marked reduction of macrophage infection is observed with more than 50% of inhibition for the highest concentrations tested (10–50 μM). S17092 appears more efficient to prevent infection than ZPP with around 50% of inhibition at 1 μM (Figures 6A,B). To investigate if the decrease of the host cell infection resulted from the POPLi inhibition rather than from the host cell POP inhibition, a 1 h pre-treatment of murine macrophages with 50 μM POP inhibitors followed by washes was performed before the invasion assay. This treatment did not affect macrophage infection by *L. infantum* (Figure 6C).

Next, to evaluate if the involvement of POPLi in the host cell invasion process would be due to extracellular interactions between POPLi and macrophage, infection assays in the presence of rPOPLi heat-inactivated or not were performed. Heat-inactivated rPOPLi or not did not alter the ability of promastigotes to infect macrophages (Figure 6D). This result was corroborated by a 30 min preincubation of promastigotes with rPOPLi antiserum, which did not inhibit macrophage infection by *L. infantum* (Figure 6E). However, when infection assay was performed in the presence of POP immune complexes (rPOPLi incubated with anti-rPOPLi serum for 30 min), significant reduction of infected cells (∼50%) was observed (Figure 6F). These results support the involvement of POPLi in host cell invasion by *L. infantum*.

## Discussion

In this work, we reported the first study of *L. infantum* prolyl oligopeptidase, focusing on its biochemical properties, cytolocalization and its involvement in murine macrophage infection by promastigotes. We showed that the best condition for active rPOPLi production was under low temperature with an induction time of 4 h, which led to shift the protein expression from an insoluble to a soluble and active form, similar to rPOPTc80 and rPOPTb (Bastos et al., 2005, 2010). rPOPLi showed five-fold increase in catalytic efficiency at pH 7.5 in the presence of reducing agent DTT in spite of the *K*_*m*_ increase in this condition, corresponding to what was already described for rPOPTc80 (Bastos et al., 2005) and rPOPTb (Bastos et al., 2010). The *k*_*cat*_/*K*_*m*_ increase may be due to the property of DTT to prevent the oxidation of Cys^255^ located near POP catalytic site (Szeltner et al., 2013), allowing higher substrate turnover, although at the same time, DTT may be interfering in the interaction between POPs and *N*-Suc-Gly-Pro-Leu-Gly-Pro-AMC, reflecting in a *K*_*m*_ increase. Despite the similar kinetic behavior of the three POPs, POPLi has a higher *K*_*m*_ (three-fold increase) than POPTc80 and POPTb (using *N*-Suc-Gly-Pro-Leu-Gly-Pro-AMC), revealing a different affinity for this substrate.

As previously described for POPTc80 and POPTb (Santana et al., 1997; Bastos et al., 2005, 2010), rPOPLi showed preference for collagenase substrates containing proline residue at position P1 of the C-terminal region, but this activity is negligible when N-terminus is free. rPOPLi has an optimal temperature at 37°C, which is in agreement with other POPs already characterized (Cunningham and O’Connor, 1998; Portugal et al., 2017) but retains only 40–50% of its enzymatic activity at 28°C. In view of the temperature difference between *Leishmania* vector and host, 28 and 37°C, respectively, POPLi optimal temperature matches well the environment for *Leishmania* growth and development inside mammalian host. Other proteases from *Leishmania* also presented optimal temperature at 37°C, including an important virulence factor for leishmaniasis, GP63 from *L. mexicana* (Chaudhuri and Chang, 1988), and a metalloprotease from *L. donovani* (Choudhury et al., 2010).

POPLi enzymatic activity was weakly inhibited by the irreversible serine protease inhibitor AEBSF (Table 1). Although AEBSF and its analog PMSF are commonly used inhibitors of serine proteases (Narayanana and Jones, 2015), they are not a bonafide POP inhibitor and this peculiarity has already been described for microorganisms such as *T. cuzi*, *T. brucei*, and *M. tuberculosis* (Santana et al., 1997; Bastos et al., 2010; Portugal et al., 2017). However, POPLi activity was strongly inhibited by TPCK and TLCK, trypsin-like and chymotrypsin-like protease inhibitors reported to inhibit POPs. ZPP and S17092, specific POP inhibitors, presented an IC_50_ value of 4.23 and 3.50 nM, respectively, comparable to that measured on POP bovine brain (López et al., 2011). This result supports the high structural similarity of POPs in the region of the catalytic pocket where ZPP or S17092 binds (Tarrago et al., 2007; Kaushik and Sowdhamini, 2011). ZPP contains a CHO group at the P1 site, which forms a hemiacetal adduct with the serine residue in the active site, mimicking the tetrahedral transition state of the enzyme catalyzed reaction (Fülöp et al., 1998) and leading to a slow-binding inhibition of POP (Bakker et al., 1990; Venäläinen et al., 2004).

**TABLE 1:** Inhibition of rPOPLi by protease inhibitors.

**Inhibitor**	**Concentration (mM)**	**Inhibition (%)**
TLCK	0.1	96.8
TPCK	0.1	92.3
PEPSTATIN A	0.1	33.5
AEBSF	10.0	19.2
EDTA	1.0	10.5
LEUPEPTIN	0.1	7.6
E-64	0.1	3.8

*Assays were performed by incubating 40 ng of rPOPLi with the inhibitor for 30 min before the addition of 20 μM of substrate. Hydrolysis was recorded up to 15 min.*

By immunofluorescence assay, POPLi was localized within the cytoplasm but also closely associated with the membrane structures including vesicles and plasma membrane. Such a subcellular localization to membranes was supported by a differential subfractionation of parasite extracts. Both POPLi and its enzymatic activity toward Suc-Gly-Pro-Leu-Gly-Pro-AMC were associated to the membrane bound protein fraction and to the cytoplasmic fraction. POPLi may be trafficked to the parasite plasma membrane and exported out of it by exosomes. It is well reported that *Leishmania* spp. release exosomes, extracellular vesicles, into their environment (Marshall et al., 2018; Pérez-Cabezas et al., 2019), which would be free to interact with host cells, either through surface binding, fusion with the plasma membrane, or by endocytosis. These vesicles may deliver effector cargos to the target cells interfering with their signaling pathways, priming them for infection (Silverman and Reiner, 2011). Proteomic analysis of *L. donovani* showed POP was present in both the cell lysate and exosomes (McCall et al., 2015). It was also shown *Leishmania* exosomes carry virulence factor candidates as GP63 isoforms, calpains, HSPs, tryparedoxin peroxidase, thimet oligopeptidase, oligopeptidase B and many others (McCall et al., 2015; Marshall et al., 2018).

As it was reported that POPTc80 inhibition reduces *T. cruzi* infection of the host cell (Grellier et al., 2001; Bastos et al., 2005), we aimed to evaluate if POPLi inhibition would have any effect in the vertebrate host cell infection. Our results showed *L. infantum* promastigote incubation with specific POP inhibitors markedly reduced macrophage infection. It is likely that the inhibitory effects of ZPP and S17092 were specific for *L. infantum* POP, as pre-treatment of macrophages with the same inhibitors did not affect the rate of infection in these cells. Also, promastigote pre-treatment with ZPP or S17092 prior to cell lysis did not affect enzymatic activity of other proteases (Figure 3E), reinforcing the specificity of these inhibitors for POP. Besides, this reduced macrophage infection by *L. infantum* was not due to parasite viability loss (Figure 3C). The involvement of POPLi in macrophage infection may not be through its direct binding to the host cell membrane, as addition of purified rPOPLi, as well as promastigote treatment with POPLi antiserum, had no effect on the infection rate. As it was reported for oligopeptidase B (Swenerton et al., 2011), it is probable that POPLi modifies, direct- or indirectly, a target on the parasite cell surface to enable its interaction with the macrophage via a specific receptor, therefore contributing for parasite entry. Of interest is the fact that rPOPLi and anti-POPLi antiserum co-incubation lead to a reduction of macrophage infection by *L. infantum* similar to that observed in the presence of specific POP inhibitors. This observation may not be due to neutralization of the enzyme activity by anti-POPLi antibodies, as neither rPOPLi alone augmented infection nor promastigote treatment with anti-POPLi antiserum inhibited macrophage infection. Thus, it can be that rPOPLi-anti-POPLi immune complexes interaction with FcRs is interfering with parasite entry into the cell. It is feasible that upon activation with immune complexes, macrophage FcRs may pair with different membrane proteins. Cell surface receptor interactions and crosstalk between signaling pathways might represent important regulatory mechanisms (Rittirsch et al., 2009; van Egmond et al., 2015; Koenderman, 2019), influencing parasite entry into the cell.

## Conclusion

Our experimental data unveil the biochemical properties of POPLi, evidencing it is present in parasite cytoplasm associated with vesicles and possibly with promastigote plasma membrane, suggesting that POPLi may be exported by exosomes out of the cell. Knowledge of potential POPLi targets may contribute to a better understanding of the role played by this protease in vertebrate macrophage infection. This study provides evidence that POPLi has potential as candidate for drug design aiming at therapy against *L. infantum* infection.

## Data Availability Statement

The datasets generated for this study are available on request to the corresponding author.

## Ethics Statement

The animal study was reviewed and approved by Animal Ethics Committee from the University of Brasília (27764/2016).

## Author Contributions

IB, JS, and PG conceptualized the project. CL, CSA, IS, FM, SC, and MG performed testing. CL, CSA, CNA, MA, and IB analyzed the data. All authors wrote the manuscript.

## Conflict of Interest

The authors declare that the research was conducted in the absence of any commercial or financial relationships that could be construed as a potential conflict of interest.
